# Mechanical behavior of CFRP partially confined coal cylinders under uniaxial compression

**DOI:** 10.1371/journal.pone.0319491

**Published:** 2025-03-10

**Authors:** Qingwen Li, Ling Li, Chuangchuang Pan, Yuqi Zhong, Fanfan Nie, Hao Yang

**Affiliations:** School of Civil and Architectural Engineering, Liaoning University of Technology, Jinzhou, China; University of Vigo, SPAIN

## Abstract

The loss of bearing capacity in abandoned coal pillars within air-mining areas is prone to cause surface settlement issues, which poses a serious threat to the safety of surface buildings. This paper thoroughly investigates the mechanical behavior of carbon fiber reinforced plastic (CFRP) partially-confined coal cylinders under uniaxial compression, aiming to explore a cost-effective technology for reinforcing coal pillars. The influence of CFRP strips on the axial compression performance of coal cylinders was systematically analyzed by adjusting two parameters: the net spacing ratio and the number of CFRP strip layers. The study shows that CFRP strip partially-confined coal cylinders and fully-confined coal cylinders exhibit similar mechanical properties, and the failure of partially-confined coal cylinders is mainly characterized by the fracture of CFRP strips and the localized fracturing of the coal cylinder. As the net spacing ratio decreases and the number of CFRP layers increases, the peak strength and deformation capacity of the coal cylinders are significantly improved, with the maximum enhancement rate reaching up to 409.36%. Under different confining conditions, the energy evolution pattern of CFRP-confined coal cylinders is generally consistent, with energy primarily accumulating in the form of elastic energy prior to reaching peak strength, and dissipated energy increasing sharply after peak strength is reached. Considering factors such as equivalent thickness, the amount of CFRP material used, and a comprehensive evaluation of economic benefits and performance enhancement, the optimal solution was identified as the CFRP confinement with a net spacing ratio of 0.25 and six layers of wrapping, offering the most cost-effective option.

## Introduction

Rich reserves, stable supply, and relatively low mining costs make coal a crucial energy resource that underpins the development of the global economy [1–2]. During the process of deep coal mining, as the mining depth increases, coal pillars often exhibit insufficient stability underground pressure [3–5]. The stability of coal pillars is closely linked to mine safety and production efficiency. Therefore, developing effective reinforcement methods to enhance the bearing capacity and resistance to destruction of coal pillars has become an urgent technical challenge that requires resolution.

At present, the commonly used reinforcement methods both domestically and internationally mainly include bolt support [6–7], grouting [8–9], shotcrete [10], and roadway-pillar combined reinforcement [11]. However, with the increasing mining depth and ground pressure, existing reinforcement technologies fail to effectively ensure the reasonable width of coal pillars, maintain internal integrity, avoid pollution, and minimize roadway space occupation, which severely impacts the mining efficiency and safety of mining areas [12]. Due to their superior mechanical properties and excellent corrosion resistance, fiber-reinforced composites have emerged as a potential material for reinforcement engineering and are now widely applied in civil engineering projects [13–16]. In 2017, Das AJ et al. [17] were the first to explore a new method of using fiber-reinforced composite materials to reinforce coal pillars and conducted uniaxial compression tests on small-sized coal pillar specimens wrapped with CFRP. The experimental results demonstrated that this method significantly improved the bearing capacity and deformation capacity of coal pillars. Liu et al. [18] investigated the axial compression performance of CFRP-confined sand-based material columns, highlighting its potential advantages in stability control for coal mine surrounding rock. Li Q et al. [19–20] observed that CFRP confinement altered the failure mode of the coal cylinder and significantly enhanced its axial deformation capacity and peak strength through axial compression tests on CFRP-confined coal cylinders. Additionally, the three-dimensional FLAC-PFC coupling analysis method was used to determine the optimal number of CFRP layers required for wrapping. Xia Z et al. [21] performed uniaxial compression and acoustic emission experiments on CFRP-confined coal cylinders and found that increasing the number of CFRP layers can alter the crack propagation mode of the coal cylinder and enhance its strain-softening characteristics during the unstable crack propagation stage. A damage constitutive model was proposed to describe the mechanical behavior of CFRP-confined coal cylinders. Feng G et al. [22] demonstrated that CFRP cloth can prevent the expansion of interface cracks and enhance the stability of the interface through splitting tests on coal-filled body composites wrapped with CFRP cloth at different interface angles.

An analysis of existing research reveals that the use of CFRP sheets to reinforce coal pillars can significantly enhance their bearing capacity and effectively improve their deformation resistance, thereby prolonging the service life of the coal pillars. However, in practice, the uncertainties of the underground environment and the presence of high residual columns significantly complicate construction operations, making the full-scale wrapping of the structure with CFRP extremely challenging. Therefore, a partial wrapping approach using FRP strips applied from the bottom up emerges as an innovative reinforcement strategy with promising applications. This method not only effectively enhances the load-carrying capacity of critical structural components through partial reinforcement technology but also minimizes interference with the normal functioning of the structure. As a result, it achieves a balance between engineering requirements and construction conditions. Compared to the traditional full wrapping method, the use of partially wrapped FRP strips presents significant advantages. Their flexibility enables them to conform to various complex structural geometries, while also greatly reducing the challenges and operational difficulties associated with environmental limitations or spatial constraints during construction. Consequently, this strategy provides a practical and efficient solution for reinforcing intricate underground structures. In this paper, the deformation and failure mechanisms of CFRP partially-confined coal cylinders under uniaxial compression are studied through experiments. The objective is to propose a more economical and effective reinforcement design method, which can not only improve the safety level of mine goafs but also reduce safety accidents caused by unstable coal pillars. This research is highly significant for improving the economic benefits of deep coal mines and promoting the sustainable development of the coal mining industry.

## 1. Experimental program

### 1.1 Coal sample preparation

**Fig 1:**
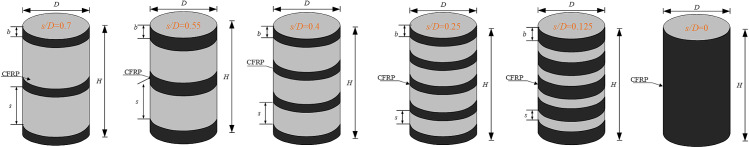
Specimen schematic.

The coal cylinders used in this study were sourced from the Tashan Coal Mine of the Datong Coal Mine Group. In accordance with the standardized test procedures of the International Society of Rock Mechanics, the coal cylinders were processed into cylindrical specimens with dimensions of 50 mm in diameter and 100 mm in height (D × H). This study utilizes CFRP cloth reinforcement technology, widely applied in the field of concrete [23]. The wet layup method was employed to wind CFRP strips or sheets around the circumference of the coal cylinders, after which the specimens were left to cure for 14 days under laboratory conditions. The lap length of the anchored CFRP composite was set at 50 mm. The experimental design includes 39 coal cylindrical specimens with different wrapping schemes, consisting of 3 unconstrained coal cylindrical control samples and 36 CFRP-confined coal cylindrical samples. The latter is further subdivided into 30 partially-wrapped specimens with CFRP strips and 6 completely-wrapped specimens with CFRP sheets. Table 1 provides the details of the specimens. In the framework of this study, the selected net spacing ratio (*R* =  s/D) is set to values less than 1. Specifically, when *R* =  0, it is regarded as a special case of CFRP fully-wrapped confined coal cylinders. Additionally, the test variables include the number of CFRP layers (1 layer, 2 layers, 3 layers) to investigate their impact on the mechanical properties of coal cylinders. The wrapping method is illustrated in Fig 1.

**Table 1:** Test scheme of CFRP-confined coal cylinder.

Specimen	*s*/mm	*L*/layers	*n*	*b*/mm
SC	/	0	3	/
S-1-0.7	35	1	2	10
S-1-0.55	27.5	1	2	15
S-1-0.4	20	1	2	10
S-1-0.25	12.5	1	2	10
S-1-0.125	6.25	1	2	15
S-1-0	0	1	2	100
S-2-0.7	35	2	2	10
S-2-0.55	27.5	2	2	15
S-2-0.4	20	2	2	10
S-2-0.25	12.5	2	2	10
S-2-0.125	6.25	2	2	15
S-2-0	0	2	2	100
S-3-0.7	35	3	2	10
S-3-0.55	27.5	3	2	15
S-3-0.4	20	3	2	10
S-3-0.25	12.5	3	2	10
S-3-0.125	6.25	3	2	15
S-3-0	0	3	2	100

### 1.2 Test equipment and test scheme

**Fig 2:**
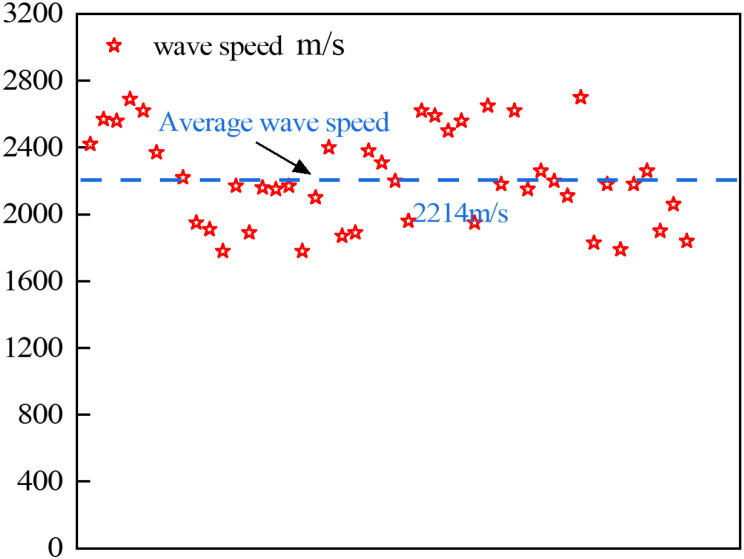
Wave velocity test of coal cylinder.

Before the test, the coal cylinders were screened and evaluated according to GB/T 50266-2013: Engineering Rock Mass Test Method Standard [24], which included a detailed inspection of their appearance, density measurement, and water content analysis. The density of the coal cylinders was found to range between 1400 and 1600 kg/m³, while their water content ranged from 3.41% to 5.82%. The NM-4A non-metallic ultrasonic testing analyzer, manufactured by Beijing Concordia, was used to measure the wave velocity of the samples. To ensure the reliability and accuracy of the test results, Vaseline coupling was applied between the probes to measure the zero sound. Subsequently, the wave velocity test was performed on the coal cylinders, as shown in Fig 2.

CFRP was applied to the coal cylinders using a two-component high-performance binder. Component *A* is a low-viscosity, high-performance epoxy resin, while Component *B* is a curing agent. The mass ratio of Component *A* to Component *B* is 2:1, and the basic parameters of the binder are provided in Table 2.

**Table 2:** Mechanical properties of impregnant.

Type	Tensile Strength/MPa	Elastic modulus/MPa	Elongation ratio/%	Compressive strength/MPa
A	≥40	≥2500	≥1.5	≥70
B	≥30	≥1500	≥1.5	≥70

**Fig 3:**
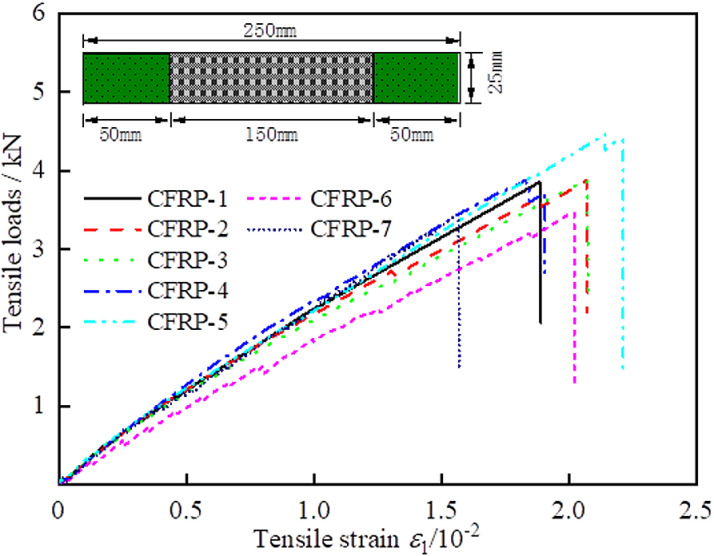
Tensile load-axial strain curves of CFRP coupons [24].

The performance parameters of the CFRP material are listed in Table 3. Based on the tensile test outlined in the “Directional Fiber Reinforced Polymer Matrix Composite Tensile Test Method,” the properties of the CFRP materials were determined [25], as shown in Fig 3. The tensile test was conducted on CFRP specimens using the WDW-300 microcomputer-controlled electronic universal testing machine, operating at a constant rate of 2 mm/min.

**Table 3:** Average mechanical properties of CFRP sheets [24].

Elastic modulus/GPa	Thickness/ mm·ply-1	Tensile strength/MPa	Ultimate tensile strain/%
47.54	0.167	918.07	1.94

**Fig 4:**
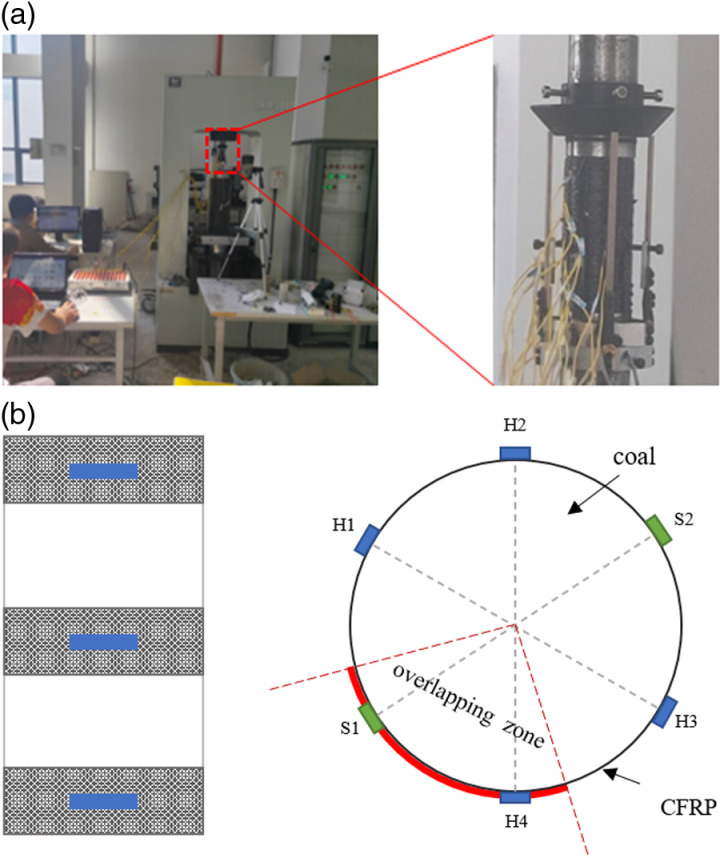
Uniaxial compression test. (a) loading test equipment (b) layout of strain gauges.

The strain and deformation of the specimen were measured using strain gauges and extensometers, as shown in Fig 4. For each confined specimen, strain gauges with a length of 20 mm were adhered circumferentially at various strip locations, and a strain gauge with the same length was installed at the center of the specimen. Specifically, there were four circumferential strain gauges spaced at 60° intervals (H1, H2, H3, H4), and two axial strain gauges spaced at 180° intervals (S1 and S2 in S1 Data). Notably, one circumferential strain gauge and one axial strain gauge were installed within the red overlap area. Additionally, two extensometers were attached to the coal sample: an axial extensometer to measure the axial displacement and axial strain, and a radial extensometer to measure the radial strain. The strains from the strain gauges were collected using the DH3816 static strain measurement system, with a sampling frequency of 50 Hz.

The uniaxial compression tests on all prepared samples were conducted using the SAM-2000 microcomputer-controlled electro-hydraulic servo rock triaxial pressure testing machine. This testing machine is capable of applying a maximum axial pressure of 2000 kN. All test data, including load, displacement, and strain, were recorded synchronously by a data recorder. Detailed information regarding the specimens is provided in Table 3. To facilitate specimen identification, ‘*SC*’ represents the unconstrained cylindrical coal sample.

## 2. Test results

### 2.1 Stress-strain relationship

**Fig 5:**
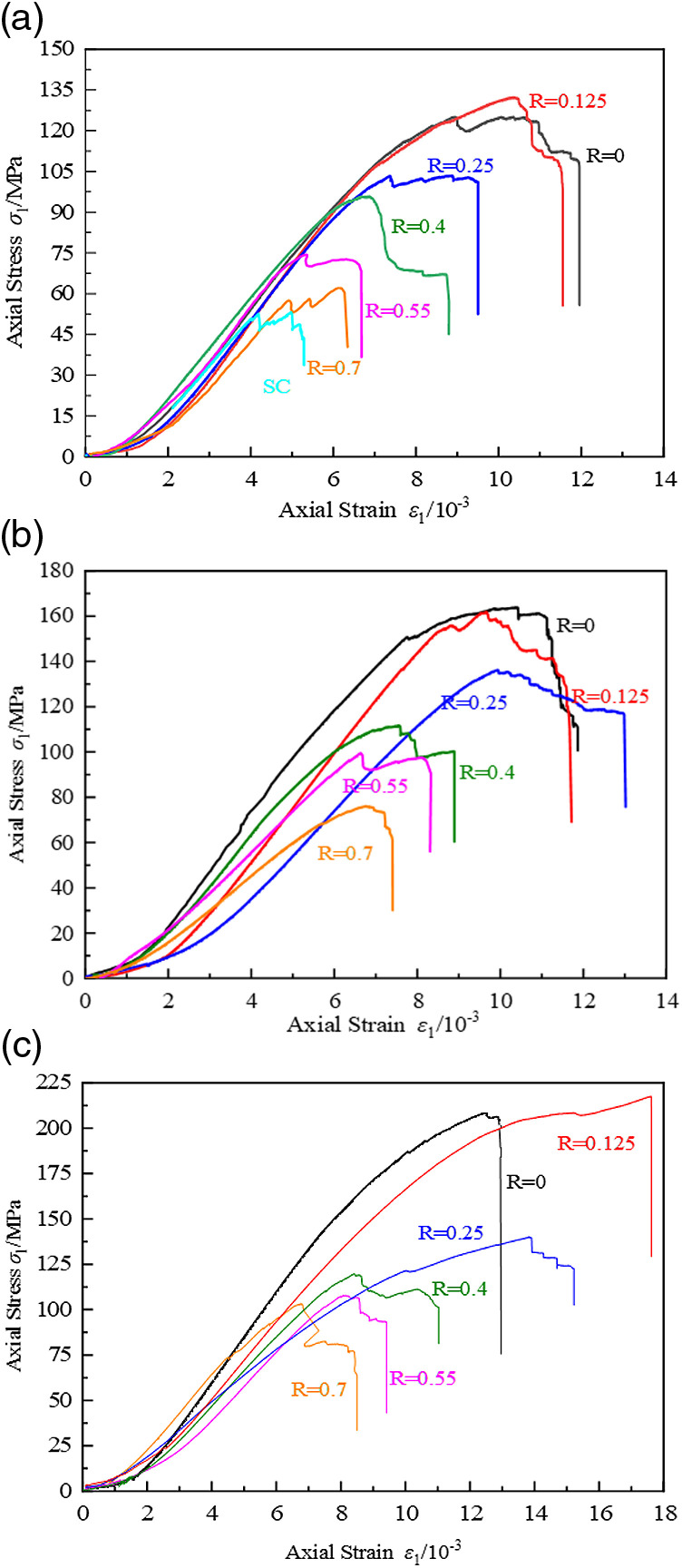
Stress-strain curves of coal cylinder with different net spacing ratios CFRP confined. (a) A layer of CFRP confined coal cylinder, (b) Two-layer CFRP confined coal cylinder, (c) Three-layer CFRP confined coal cylinder.

Fig 5 presents the stress-strain curve of the CFRP-confined coal cylinder. During the loading process leading to failure, the stress-strain curve typically undergoes four distinct stages [26]:

Compaction stage: This stage corresponds to the initial phase of loading, where the microcracks and inherent defects within the sample gradually close and compact under the influence of stress. On the stress-strain curve, this process is characterized by a short concave segment, which indicates the initial adjustment of the sample’s internal structure.

Linear elastic deformation stage: As the stress level gradually increases, the coal cylinder enters the elastic deformation stage, during which the coal cylinder primarily resists the pressure applied by the testing machine. In this stage, there is a clear linear relationship between stress and strain, reflecting the stable and predictable response of the material within the elastic range.

Elastic-plastic stage: When the stress exceeds the elastic limit of the coal cylinder, the sample enters the elastic-plastic deformation stage. At this point, the stress-strain relationship exhibits nonlinear characteristics, indicating that the specimen begins to yield and undergoes a hardening process. Although the internal microcracks begin to expand, the constraint effect of CFRP effectively slows the growth rate of strain, allowing the specimen to maintain a high bearing capacity. This results in a slow upward trend in the stress-strain curve, reflecting the hardening behavior of the specimen.

Failure stage: After reaching the peak stress, the sample enters the failure stage. During this stage, the internal cracks within the specimen expand rapidly and begin to interconnect, and the constraint efficiency of the CFRP gradually diminishes. As a result, the deformation of the specimen is no longer restricted, the strain increases sharply, and the stress decreases rapidly until the specimen is ultimately destroyed. It is worth noting that, compared to the unconstrained coal cylinder without CFRP confinement, the strain growth of the CFRP-confined coal cylinder is significantly slower during this stage, indicating a notable improvement in its initial stiffness.

The stress in the stress-strain curve of the unconstrained coal cylinder decreases rapidly after reaching the compressive strength, exhibiting typical brittle failure characteristics. In contrast, the fully-constrained coal cylinder demonstrates completely different mechanical behaviors. This difference is due to the strong constraint provided by the CFRP, which complicates the failure process of the specimen. Before reaching the compressive strength, the CFRP exerts a circumferential constraint that delays the specimen’s failure process, resulting in a stress-strain curve with a prolonged rising stage. Near the compressive strength, the CFRP begins to break, causing the specimen to lose its bearing capacity and the stress to decrease rapidly. It is noteworthy, however, that due to the CFRP constraint, the curve exhibits a “drop-up” phenomenon. This behavior arises from the interplay between the inhomogeneity and brittleness of coal materials and the restraining effect of CFRP. Coal, characterized by significant inhomogeneity and low toughness, is susceptible to crack initiation and rapid propagation when subjected to external loads, resulting in a sharp decrease in stress. Conversely, CFRP, as a high-strength and ductile material, has its restraining effectiveness gradually revealed with increasing strain. At lower stress levels, the restraining effect of CFRP has not been fully realized, leading to an initial “drop” in stress. However, as strain continues to increase, the restraining effect of CFRP on the coal column strengthens, effectively mitigating the sharp decline in stress. This results in a recovery and subsequent rise in stress levels, exemplifying the phenomena of stress concentration, cracking, and reclosure. This process reflects the dynamic interaction between stress concentration, crack propagation, and closure. In addition, as the number of CFRP layers increases, the compressive strength and ultimate strain of the specimen also increase, indicating that the confinement effect of CFRP is further enhanced. The stress-strain curve of the strip-constrained coal cylinder exhibits plastic failure characteristics similar to those of the fully-constrained coal cylinder. Compared to the unconstrained coal cylinder, the compressive strength and deformation capacity of the strip-constrained coal cylinder are significantly improved. Furthermore, it can be observed that the strain growth of the strip-confined coal cylinder is slower than that of the unconfined coal cylinder, indicating that the initial stiffness of the strip-confined coal cylinder is enhanced.

### 2.2 Failure modes

**Fig 6:**
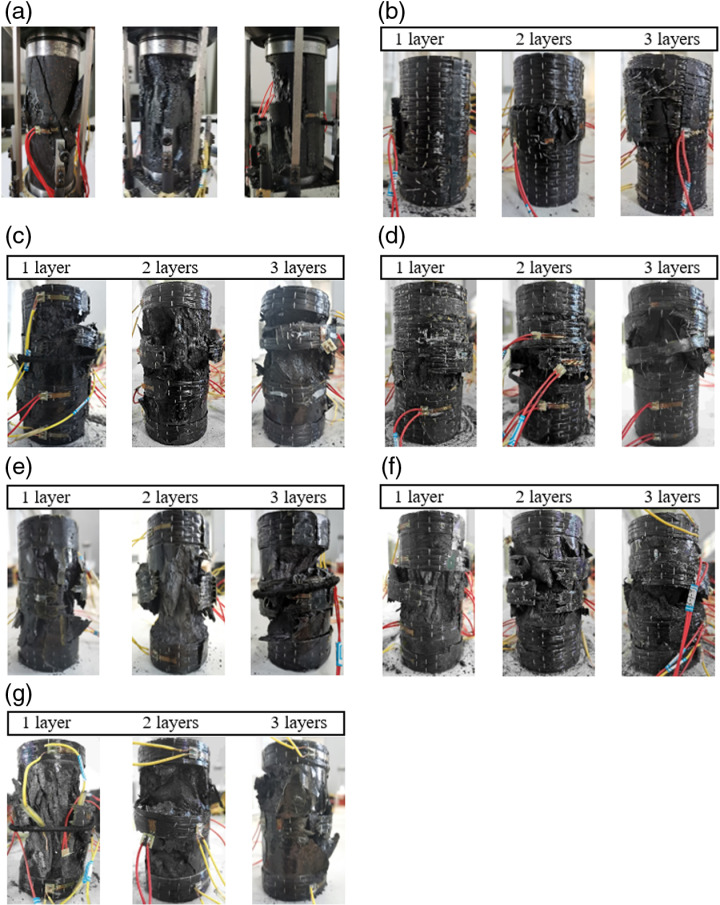
Failure mode of CFRP-confined coal cylinder with different net spacing ratios. (a) Unconfined coal sample (b) R = 0 (c) R = 0.125 (d) R = 0.25 (e) R = 0.4, (f) R = 0.55 (g) R = 0.7.

Under axial compressive loading, unconstrained coal samples develop microcracks on their surface. As the load increases, these microcracks gradually propagate, resulting in a tensile-dominated failure mode supplemented by shear failure, forming a tensile-shear mixed failure pattern. Coal fragments spall off along the cracks, and the coal sample experiences severe damage, losing its original shape entirely. In contrast, the failure of CFRP fully-constrained coal cylinders originates from the fracture of the CFRP fabric, as shown in Fig 6.

Under axial compression, the CFRP at the middle of the coal sample is subjected to circumferential tensile forces. As the axial pressure increases, the coal sample undergoes complex stress transfer and deformation behaviors. Due to the brittle nature of the coal, microscopic cracks are generated in localized areas, accompanied by certain volumetric expansion. During this process, the CFRP material, with its high strength and excellent toughness, provides circumferential confinement to the coal sample’s expansion, effectively delaying lateral deformation of the coal sample and enhancing its load-bearing capacity. However, when the circumferential expansion stress of the coal sample exceeds the tensile strength limit of the CFRP, the material undergoes tensile failure. This scenario is typically characterized by the rapid propagation of internal cracks in the coal sample and the instantaneous release of strain energy, rendering the CFRP incapable of providing effective circumferential confinement. Once the CFRP constraint is lost, the compressive strength of the coal sample decreases significantly, and its axial load-bearing capacity rapidly diminishes, eventually leading to failure. This failure process reflects the strong circumferential constraint provided by CFRP on the coal sample’s expansion while also revealing the performance limits of CFRP under circumferential tension. When the strength of the CFRP is insufficient to resist the expansion stress generated by the coal sample, the entire system inevitably becomes unstable, causing the coal sample to completely lose its axial load-bearing capacity and fail.

For the CFRP strip-partially confined coal cylinder, the failure mode exhibits two characteristics: the fracturing of the CFRP strip and localized breakage of the coal cylinder. The fracture phenomenon of CFRP strips often occurs in the middle of the coal cylinder. As axial compression is applied, the coal cylinder expands outward, causing the CFRP ring to be subjected to tensile forces. When the circumferential tensile strength of the CFRP strip is insufficient to withstand this expansion tension, a fracture occurs, resulting in a loss of axial bearing capacity of the coal cylinder. The localized fracture phenomenon of the coal cylinder is mainly concentrated in the unconfined area in the middle of the coal cylinder. Due to the limited constraint in this area, the ultimate compressive stress is relatively low. When subjected to axial load, the section of the coal cylinder between the CFRP strips experiences tensile deformation, while the coal cylinder near the CFRP is subjected to stress concentration, leading to breakage. This breakage contributes to the fracturing of the CFRP and accelerates the failure of the coal cylinder in that region.

The interaction of the two failure mechanisms jointly determines the final failure mode of the coal cylinder. The fracture of the CFRP strips creates conditions for the localized fracture of the coal cylinder, while the localized fracture of the coal cylinder further weakens the constraining capacity of the CFRP strips. The net spacing ratio, as a key parameter, regulates the interaction between these two failure mechanisms, thereby directly affecting the overall stability and failure mode of the system. The net spacing ratio is a critical parameter in determining the constraining strength of the CFRP strips on the coal cylinder. When the net spacing ratio is small, the CFRP strips are distributed more densely, exerting a more significant circumferential confinement effect on the expansion of the coal cylinder. This confinement effectively delays internal stress concentration and crack propagation within the coal cylinder, thereby enhancing the overall axial load-bearing capacity of the structure. However, as the net spacing ratio increases, the unconfined regions between the CFRP strips expand, resulting in more pronounced expansion of the coal cylinder in these regions, which aggravates the phenomenon of localized stress concentration. The localized expansion stress exceeds the circumferential tensile limit of the CFRP strips more quickly, leading to strip fracture and accelerating the fracture behavior of the coal cylinder in the unconfined regions.

A closer look reveals that while increasing the number of CFRP layers can enhance the constraining effect, it can also exacerbate the failure of the coal cylinder. An increase in layer count restricts the lateral expansion of the coal cylinder, leading to heightened internal stress concentration. Once the strength limit of the coal cylinder is surpassed, it results in severe fracture. Furthermore, the net spacing ratio plays a crucial role in influencing both the failure mode and mechanical behavior of the coal cylinders. When the net spacing ratio is excessively large (*R* ≥  0.4), the overall constraining effect of the CFRP on the coal cylinder is diminished, resulting in significant spalling of the coal cylinder [27]. Although the widening of strip spacing reduces the constraint efficiency of the CFRP, it still exhibits superior strength and deformation capability compared to an unconstrained coal cylinder. In contrast, when the net spacing ratio is moderate or small (*R* <  0.4), the CFRP strips can effectively constrain the coal cylinder and restrict its lateral deformation. Under these conditions, the failure mode is primarily characterized by local spalling, and the area of spalling diminishes as the net spacing ratio decreases. Ultimately, the failure is governed by the fracture of the central CFRP strips, which underscores the crucial role of these strips in controlling the failure of coal cylinders.

## 3. Ultimate condition

### 3.1 Ultimate axial stress

**Fig 7:**
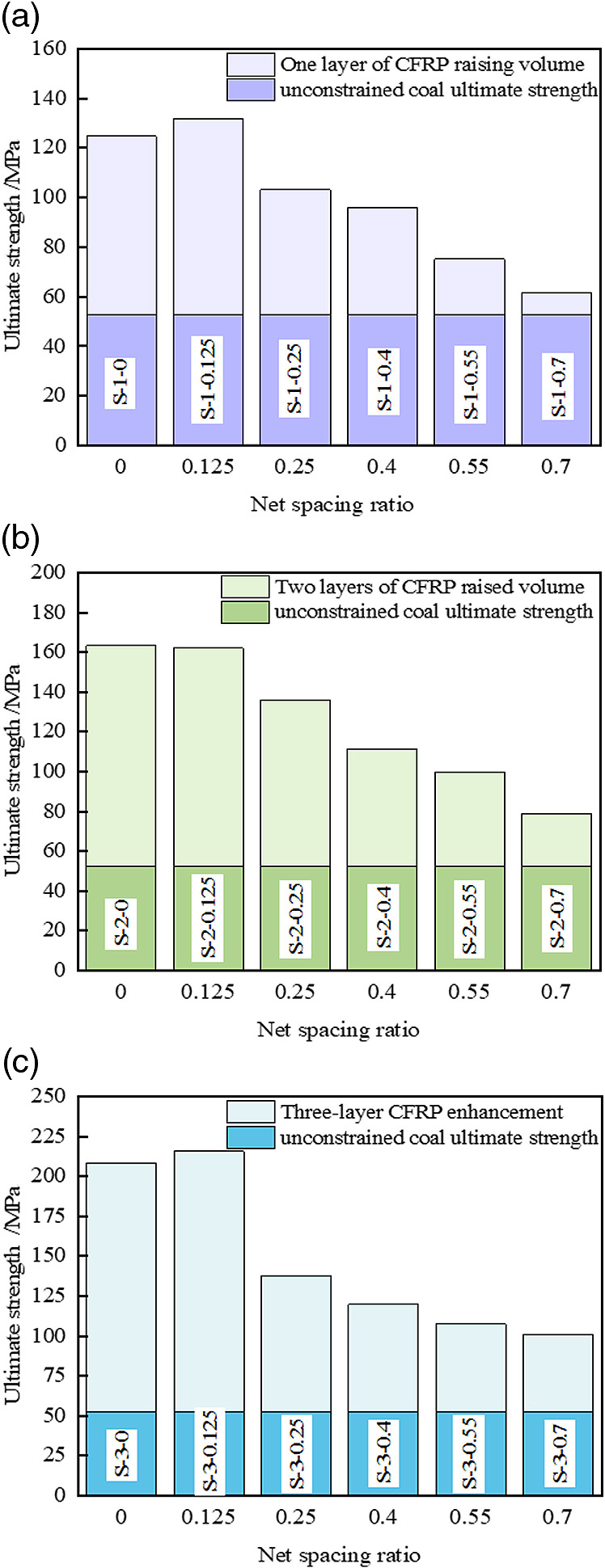
Enhancement of ultimate axial stress. (a) A layer of CFRP confined coal cylinder (b) Two-layer CFRP confined coal cylinder (c) Three-layer CFRP confined coal cylinder.

The net spacing ratio and the number of layers of CFRP have a significant effect on the ultimate strength of the specimen. As illustrated in Fig 7, the overall trend of the histogram demonstrates a consistent downward pattern. Compared with the unconfined coal cylinder, the compressive strength of the CFRP strip-confined coal cylinder is significantly enhanced, showing increases of 17.03%, 49.83%, and 91.32%, respectively, for *R* =  0.7. For *R* =  0.55, the compressive strength increased by 42.59%, 89.09%, and 103.91%, respectively. Similarly, for *R* =  0.4, the strength increased by 82.34%, 111.41%, and 127.23%. At *R* =  0.25, it rose by 95.75%, 157.56%, and 161.93%, respectively. Finally, for *R* =  0.125, the compressive strength exhibited substantial increases of 150.11%, 207.59%, and 309.36%, respectively. Under the condition of a constant number of layers, the ultimate strength of the CFRP partially confined coal cylinder declines as the net spacing ratio increases. This phenomenon can be attributed to the fact that an increase in the net spacing ratio reduces the overall fiber volume ratio of the specimen, thereby weakening the confining stresses. Additionally, an increase in the net spacing ratio exacerbates the arching effect of the strip constraint, further reducing the constraint efficiency. Conversely, a smaller net spacing ratio results in a more continuous constraint, which enhances the ultimate strength by minimizing the strip spacing. Therefore, a rational design of the net spacing ratio is critical for optimizing the mechanical properties of CFRP-confined columns.

When the net spacing ratio is fixed, an increase in the number of CFRP layers (from 1 to 3 layers) significantly enhances the confining effect, resulting in a corresponding increase in the ultimate strength of the coal cylinder. Specifically, when *R* =  0.125, the ultimate strength of the coal cylinder with three layers of CFRP is 63.67% and 33.09% higher than that of the one-layer and two-layer configurations, respectively. Similarly, when *R* =  0.7, the ultimate strength increases by 64.48% and 27.69%, respectively. The increase in the number of CFRP layers directly augments the overall thickness and confining capacity of the reinforcement material, effectively limiting internal crack propagation and delaying the failure process. This, in turn, improves the overall bearing capacity of the specimen. It is worth noting that under certain conditions (S-2-0.125 and S-3-0.125), the ultimate strength of the strip-constrained coal cylinder approaches that of the fully constrained coal cylinder. However, the strip-constrained configuration proves to be more economical and efficient in material usage. Notably, when the number of layers is 1 or 3, the ultimate strength even surpasses that of the fully constrained configuration. This observation highlights the significant cost and performance advantages of the strip constraint in engineering applications.

**Fig 8:**
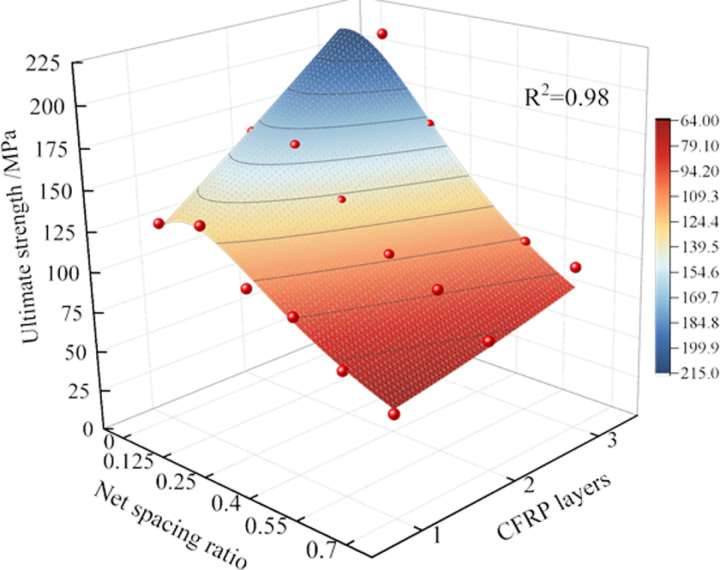
3Dsurface of peak strength of CFRP-confined coal cylinder.

In summary, both *L* and *R* exert a significant positive influence on the compressive strength (*f*_cu_) of the coal cylinder. These two parameters are interdependent, and their combined influence leads to a substantial improvement in the bearing capacity. To further investigate the relationship between *f*_cu_, *R*, and *L*, a study was conducted based on the variation law of the peak strength of CFRP-confined coal cylinders across different layers. Following the principles of high fitting accuracy, simplicity in functional form, and minimal parameters, the nonlinear fitting model comparison function in Origin software was employed. Through this screening process, a general response function was obtained, which effectively characterizes the evolution law of the peak strength of CFRP-confined coal cylinders under different layers, as presented in Eq. (1):


$$f_{cu}=\frac{89.505+86.998\times R+26.723\times L}{1-0.61\times R+9.392\times R^{2}-7.475\times R^{3}-0.064\times L}$$ (1)

where, *f*_cu_ is the peak strength of CFRP confined coal cylinder, MPa. The response function surface is shown in Fig 8.There is a high correlation between the surface and the experimental data (R^2^ =  0.98).

### 3.2 Ultimate strain

**Fig 9:**
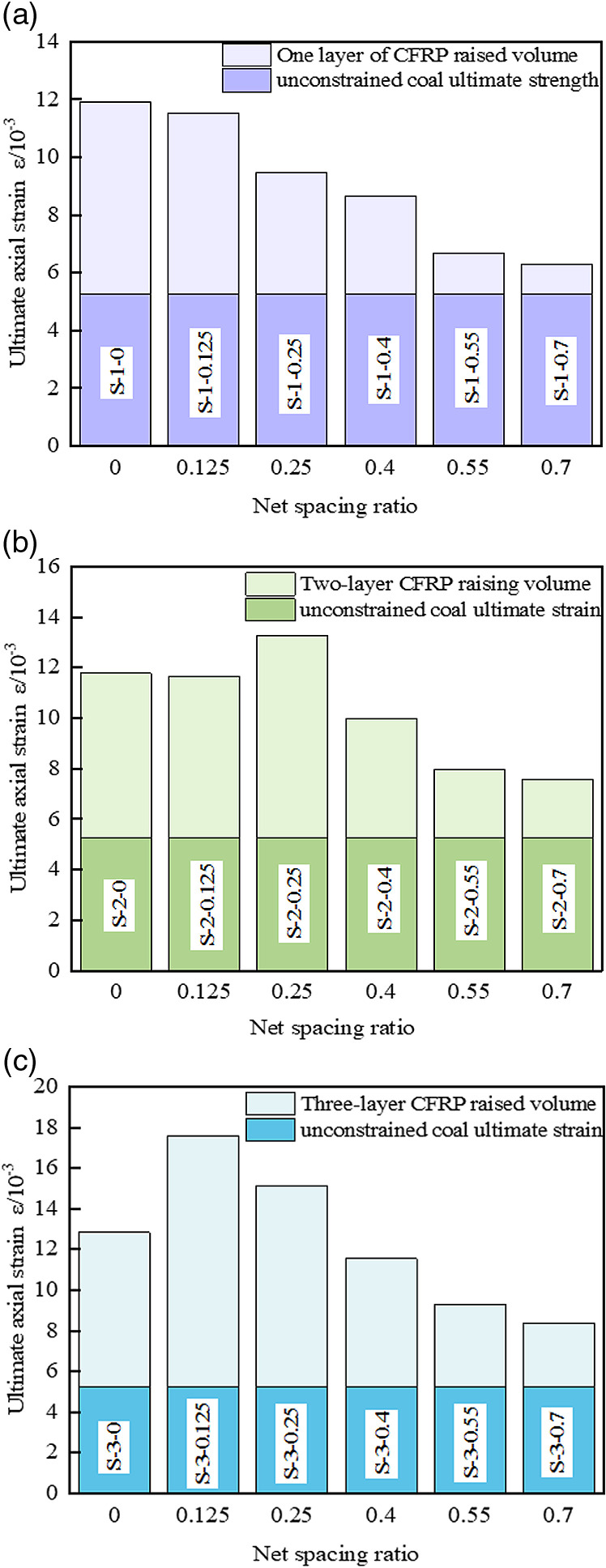
Enhancement of ultimate axial strain. (a) A layer of CFRP confined coal cylinder (b) Two-layer CFRP confined coal cylinder (c) Three-layer CFRP confined coal cylinder.

The net spacing ratio of CFRP and the number of layers are the primary control factors influencing the ultimate strain of the specimen. As shown in Fig 9, the ultimate axial strain of CFRP-confined coal cylinders generally exhibits a downward trend with increasing net spacing ratio, reaching its lowest value at *R* =  0.7. Compared with the unrestrained coal cylinder, the ultimate strain of CFRP strip-confined coal cylinders under different layers is notably improved: when *R* =  0.7, it increases by 19.58%, 43.92%, and 59.32%, respectively; when *R* =  0.55, it increases by 26.99%, 51.33%, and 76.62%, respectively. When *R* =  0.4, the ultimate strain increases by 64.56%, 89.92%, and 119.2%, respectively; for *R* =  0.25, the increases are 80.23%, 152.47%, and 187.42%, respectively. Finally, when *R* =  0.125, the ultimate strain increases by 119.11%, 121.48%, and 234.41%, respectively. Specifically, a smaller net spacing ratio indicates that the CFRP strips are more closely spaced, which enhances their continuous and tight constraint on the specimen. This mechanism of tight constraint effectively suppresses crack propagation and local material failure, thereby increasing the ultimate strain capacity of the specimen. Conversely, an increase in the net spacing ratio results in wider strip spacing, which weakens the constraint efficiency.

Increasing the number of CFRP layers significantly enhances the ultimate strain of the specimen, primarily due to the increased binding force provided by the multi-layer structure. This enhanced binding effect not only expands the plastic deformation zone of the sample but also improves the material’s ductility under loading conditions, effectively suppressing crack propagation. Such a mechanism allows the material to sustain greater strain beyond the peak stress before fracture, thereby enhancing its overall bearing performance. Under a constant net spacing ratio, the axial strain of CFRP is positively correlated with the number of layers, meaning that the strain increases as the number of layers increases. For instance, the ultimate axial strain of the three-layer CFRP strip partially wrapped coal cylinder (S-3-0.125) is 37.21% higher than that of the fully constrained configuration, highlighting the substantial enhancement in lateral confinement and deformation capacity provided by the CFRP strip.

**Fig 10:**
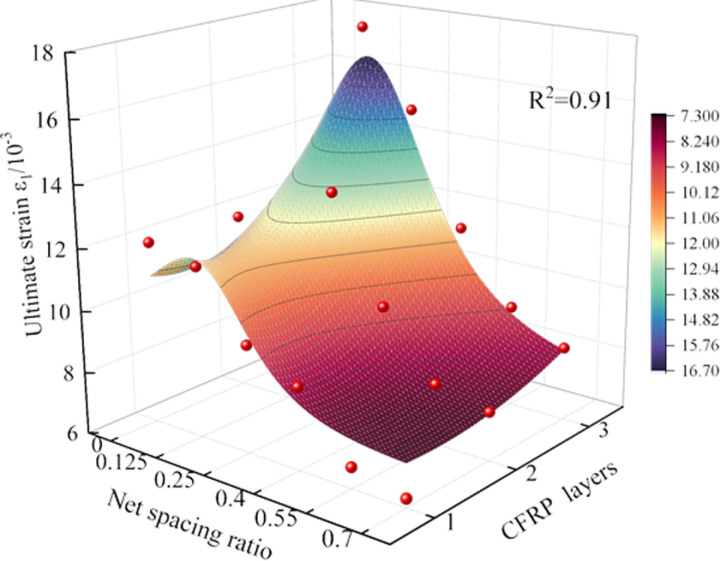
3Dsurface of ultimate strain of CFRP-confined coal cylinder.

Based on the *ε* distribution law of the CFRP-confined coal cylinder under varying layers, a rational function is proposed to characterize the general response function, which incorporates the effect of *R* under different wrapping layers, as shown in Eq. (2):


$$\varepsilon =\frac{14.474-54.305\times R+147.424\times R^{2}-4.152\times L+1.986\times L^{2}}{1-6.148\times R+19.242\times R^{2}-0.135\times L}$$ (2)

where, *ε* is the ultimate axial strain. The response function surface is shown in Fig 10 (R^2^ =  0.91), and the fitting accuracy is high and the effect is significant, indicating that the formula has certain reliability.

### 3.3 Hoop strain

**Fig 11:**
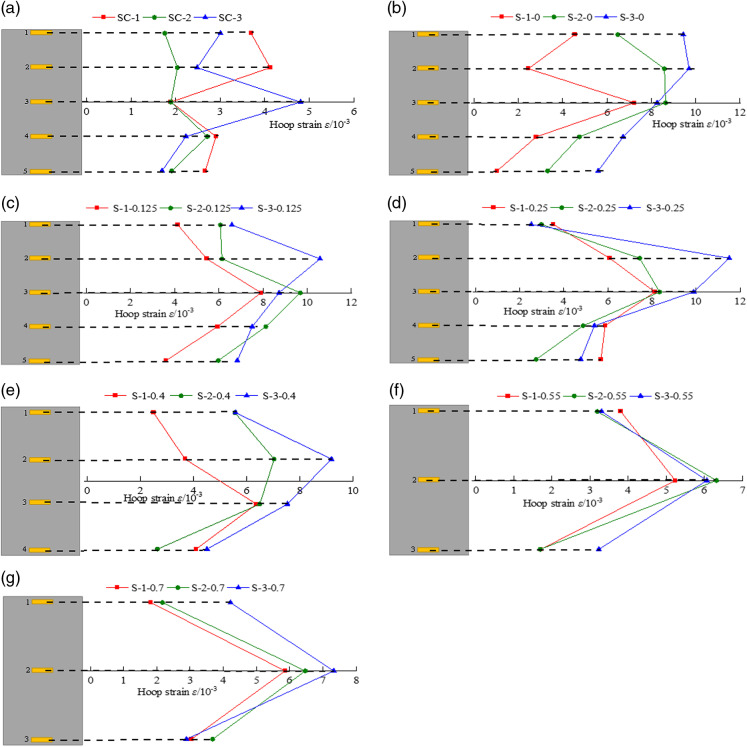
Circumferential strain at different heights of coal samples. (a) Unconfined coal sample (b) R = 0 (c) R = 0.125 (d) R = 0.25 (e) R = 0.4 (f) R = 0.55 (g) *R* = 0.7.

In order to more intuitively compare the circumferential strain of CFRP strip-constrained coal cylinders at different heights under varying net spacing ratios, the circumferential strain at the peak stress point is analyzed. The numbers 1, 2, 3, 4, and 5 in Fig 11 correspond to the strain gauges positioned at different heights.

Fig 11(a) illustrates the circumferential strain of unconstrained coal cylinders at different heights. It can be observed that the ultimate circumferential strain varies at different heights. This is primarily due to the heterogeneity and anisotropy within the coal cylinder, which leads to an uneven distribution of internal stress under external loading. As a result, micro-defects and cracks within the coal cylinder further propagate and evolve during the loading process, contributing to the inhomogeneity of the circumferential deformation.

Fig 11(b) presents the circumferential strain of the fully constrained coal cylinder at different heights. Under the influence of external load, the circumferential strain exhibits a characteristic variation, being larger in the middle and upper sections and smaller at the bottom. This behavior results from the interaction between the coal cylinder and CFRP. Specifically, when the load is applied to the top of the coal cylinder, the middle and upper regions initially experience significant stress, leading to a larger circumferential strain. In the bottom region, due to the delayed stress transfer and the constraint imposed by the CFRP, the stress in the coal cylinder is lower, resulting in smaller hoop strain. The diagram also reveals that the circumferential deformation is significantly enhanced compared to that of the unconstrained coal cylinder. During the loading process, the constraint effect of CFRP gradually becomes more pronounced as the coal cylinder deforms, and the circumferential deformation is controlled within a certain range due to its high stiffness characteristics. Since CFRP is uniformly distributed across the surface of the coal sample, the constraint effect on the coal cylinder is also relatively uniform. However, due to the boundary effect and stress concentration in the upper region, the circumferential strain in this area is relatively larger.

Fig 11(c), (d), (e), (f), and (g) show the circumferential strain diagrams of the CFRP strip-constrained coal cylinder, which differs from that of the fully CFRP-constrained coal cylinder. These diagrams exhibit the characteristic variation of large strain in the middle and smaller strain at both ends. This is primarily due to the fact that, under the constraint of CFRP strips, the influence of heterogeneity within the coal cylinder on the circumferential strain distribution is reduced. As a result, the stress distribution inside the coal cylinder becomes more uniform, with the central region acting as the main area for stress concentration and strain increase. Since the CFRP strips are spaced discontinuously along the side of the coal cylinder, the deformation in the strip-constrained areas will be limited to some extent during the loading process. However, because the regions between the strips are unconstrained, the circumferential deformation in these areas is relatively larger. Therefore, the circumferential strain of the coal cylinder constrained by CFRP strips exhibits the characteristic variation of large strain in the middle and smaller strain at both ends.

## 4. Energy consumption analysis

### 4.1 Analysis of energy evolution stage

The coal cylinder undergoes the storage and release of energy from loading to failure. When the external load is applied to the sample, part of the energy is converted into elastic energy, which is the elastic deformation energy of the sample. The remaining energy is dissipated in the form of cracks, friction, and microstructural changes. This conversion of energy is referred to as damage. As the load increases, damage accumulates continuously, eventually leading to the failure of the coal cylinder. It is assumed that the coal cylinder, under external load, remains in an ideal state without heat exchange during the process of physical deformation. The relationship with the first law of thermodynamics is as follows:


$$U=U^{d}+U^{e}$$ (3)

where, *U* is the total input energy of the deformation process, *U*^d^ is the dissipation energy dominated by plastic energy and damage energy, and *U*^e^ is the elastic energy stored in the coal cylinder.

**Fig 12:**
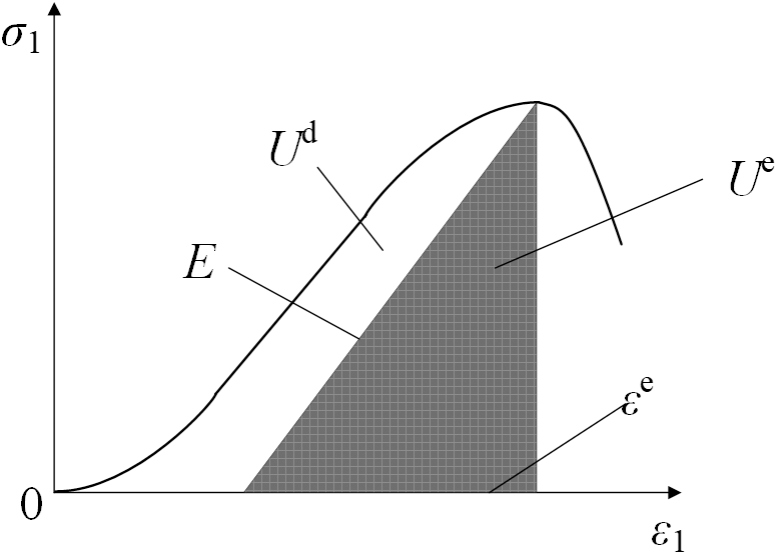
Relationship between elastic and dissipative energy.

When the elastic energy accumulates to a certain extent and the dissipative damage reaches a critical limit, the CFRP-confined coal cylinder will lose its stability and release its internal energy. The relationship between the elastic strain energy and the dissipated energy of the CFRP-confined coal cylinder is illustrated in Fig 12.

The total energy of coal cylinder is [28]：


$$U=\int_{0}^{\varepsilon_{1}}\sigma_{1}d\varepsilon_{1}+\int_{0}^{\varepsilon_{2}}\sigma_{2}d\varepsilon_{2}+\int_{0}^{\varepsilon_{3}}\sigma_{3}d\varepsilon_{3}$$ (4)

For uniaxial compression, where *σ*₂ =  *σ*₃ =  0, Huang [29] investigated the feasibility of using the initial elastic modulus instead of the unloading elastic modulus. Theoretical verification indicates that this calculation method is valid. The calculation formula for elastic energy can be simplified as follows:


$$U^{e}=\frac{\sigma_{1}^{2}}{2E}$$ (5)

According to the concept of area calculated by definite integral, the calculation formula of total energy *U* is:


$$U=\sum_{i=1}^{n}\frac{\left(\sigma_{1}^{i}+\sigma_{1}^{i+1})(\varepsilon_{1}^{i}-\varepsilon_{1}^{i+1}\right)}{2}$$ (6)

where, *σ*_1_^i^ is the stress at any point of the axial stress-strain curve of CFRP confined coal cylinder, MPa; *ε*_1_^i^ is any point strain in the stress-strain curve, %.

**Fig 13:**
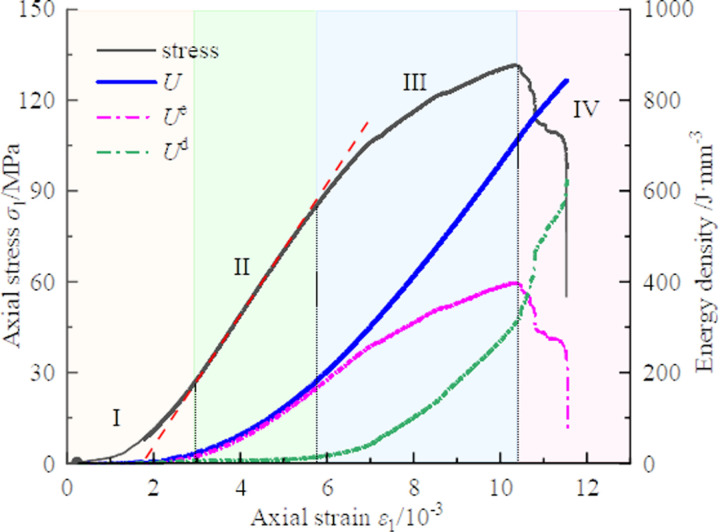
Energy evolution curves during deformation and failure of a typical CFRP-confined coal cylinder(S-1-0.125).

Based on the characteristics of energy evolution, the damage and fracture process of coal cylinders can be divided into four stages [30,31], as shown in Fig 13: the crack compaction stage, the elastic deformation stage, the unstable damage accumulation stage, and the post-peak failure stage.

During the crack compaction stage, the primary microcracks, pores, and defects within the coal cylinder are gradually compacted and closed under increasing stress, accompanied by a reduction in void volume and an increase in dissipated energy. This process enhances the overall compactness and initial stability of the coal cylinder, establishing the foundation for subsequent stress loading.

During the elastic deformation stage, the coal cylinder exhibits linear elastic characteristics, meaning that stress is proportional to strain. In this period, the coal cylinder can effectively bear the external stress without significant damage, and the input energy is primarily converted into elastic energy and stored within the body. At this stage, dissipated energy remains constant, as all energy is dedicated to elastic deformation, with no new crack formation occurring.

The unstable damage accumulation stage marks the onset of nonlinear behavior in the coal cylinder. With the continuous application of stress, micro-crack initiation and damage accumulation lead to stiffness degradation and the initiation of plastic deformation. The mechanical properties of the coal cylinder gradually deteriorate, though the critical point of fracture has not yet been reached. During this stage, the formation and development of new cracks contribute to an increase in dissipated energy, reflecting the energy consumption involved in the crack propagation process.

In the post-peak failure stage, damage accumulates to the critical point, and the coal cylinder enters the accelerated damage expansion period. Micro-cracks spread rapidly and interconnect, forming macro-cracks and leading to brittle failure. During this process, elastic energy is released sharply, and dissipated energy rises rapidly, which can be attributed to the significant amount of energy required for the formation and expansion of macroscopic cracks.

These four stages describe the complete evolution process of the coal cylinder, from the initial stable state to final rupture during the loading process, as well as the processes of energy input, storage, dissipation, and release. Each stage reflects changes in the internal structure and mechanical properties of the coal cylinder. Understanding these stages is crucial for predicting the fracture behavior of coal cylinders and for designing appropriate reinforcement measures.

### 4.2 Analysis of energy dissipation ratio

As the core support and pressure control structure in the coal mining system, the stability of the coal pillar plays a decisive role in the safety of mining operations. The failure and deformation process of coal pillars under external load represents a complex process of energy conversion and dissipation [32,33]. Based on the fundamental principle of energy conservation, the instability and failure of coal pillars induced by external load can be viewed as a continuous process of energy input, accumulation, and eventual dissipation. To investigate the energy dissipation of the specimen under strip constraints, the energy dissipation ratio is employed to analyze the energy dissipation characteristics of the coal cylinder during uniaxial compression.

**Fig 14:**
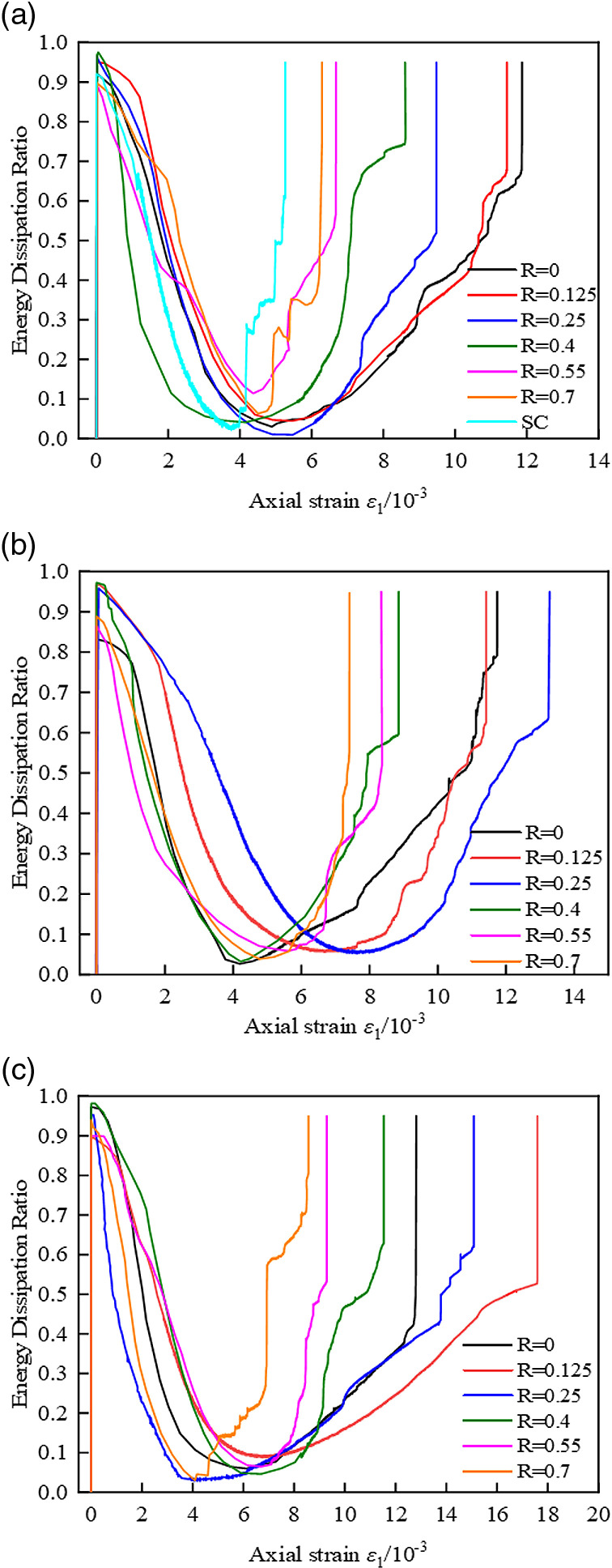
Energy dissipation ratio curves for CFRP-confined coal cylinders. (a) A layer of CFRP confined coal cylinder. (b) Two-layer CFRP confined coal cylinder (c) Three-layer CFRP confined coal cylinder.

Fig 14 shows in detail the dynamic mode of the energy dissipation ratio in relation to strain evolution during the loading process of the coal cylinder, displaying an ‘N’ trend: rising initially, then falling, and rising again. In the initial stage of loading, the input energy is primarily used to close the micro-fractures within the coal cylinder. This process is accompanied by friction and resistance at the fracture surfaces, resulting in an increase in the energy dissipation ratio compared to the initial stage. Subsequently, once fracture closure is completed, the coal cylinder enters the elastic energy storage stage. During this phase, micro-fractures develop stably, and the energy dissipation ratio decreases, approaching zero. When the stress reaches the critical value, the micro-cracks accelerate and interconnect, signaling that the coal cylinder has entered the stage of instability and failure. At this point, the proportion of dissipated energy increases sharply, energy is dissipated rapidly, and the structural integrity of the coal cylinder deteriorates significantly, ultimately leading to failure.

Compared with unconstrained coal cylinders and CFRP-confined coal cylinders, unconstrained coal cylinders are more prone to failure under external force due to their inherent physical and mechanical properties. This is manifested by a sharp increase in the energy dissipation ratio, accompanied by rapid energy release and structural collapse, which poses a significant threat to the safety of coal mine engineering. The CFRP enhances the energy absorption capacity of the coal cylinder by applying additional constraints [34], enabling the coal cylinder to absorb more energy through its own deformation, failure, and the CFRP strain energy conversion mechanism before failure, thereby improving overall bearing capacity and stability. The variation in the energy dissipation ratio of partially wrapped coal cylinders with different net spacing ratios of CFRP strips is generally similar. However, coal cylinders with a larger net spacing ratio exhibit a smaller proportion of dissipated energy during the final sharp rise. This phenomenon can be attributed to the fact that the overall constraint of CFRP on the coal cylinder is weakened under a larger net spacing ratio, preventing the coal cylinder from effectively accumulating energy at lower deformation levels, which results in earlier damage.

## 5. Discussions

### 5.1 Failure mechanism

**Fig 15:**
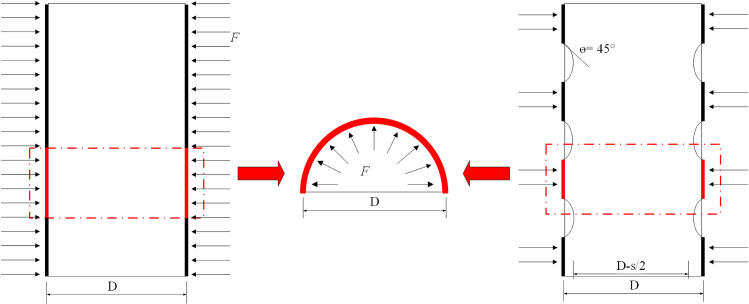
Stress state of fully and partially CFRP-confined coal cylinders [26].

Fig 15 presents the stress model diagram of the coal cylinder under different CFRP constraint forms (full constraint and strip constraint). It can be observed that when CFRP is used to constrain the coal cylinder, its high tensile strength transforms the stress state of the coal cylinder into a three-dimensional compression state [17]. This state inhibits crack propagation within the coal cylinder, delays the failure process, and enhances the bearing capacity and ductility of the coal cylinder. CFRP is highly effective in absorbing and dissipating significant amounts of energy during the damage of coal columns. As micro-cracks and internal damages within the coal column propagate, CFRP can inhibit the further expansion of these cracks while absorbing and dissipating energy through its own deformation. This mechanism of energy absorption and dissipation not only reduces the extent of damage to the coal column but also enhances its seismic performance. The test demonstrates that the compressive strength of the CFRP fully confined coal cylinder increases significantly, rising several times from 1 to 3 layers of CFRP. In the three-dimensional compression state, the ductility of the coal cylinder is improved, and its deformation capacity at failure is also enhanced. The failure mode of the CFRP fully confined coal cylinder is primarily CFRP tensile fracture, which is more ductile compared to the brittle failure of the unconfined coal cylinder. Throughout the compression process, the coal cylinder is uniformly constrained, resulting in relatively uniform crack propagation and failure mode, with no obvious localized failure.

The stress state of the coal cylinder confined by CFRP strips is non-uniform. The unrestrained part primarily experiences axial pressure, while the restrained part is subjected to three-dimensional pressure. This condition is prone to stress concentration and local damage. The constraint effect of CFRP strips on the coal cylinder increases as the net spacing ratio decreases. The failure mode includes CFRP tensile fracture and crushing of the unrestrained part. Under the constraint of CFRP strips, crack propagation and failure modes exhibit distinct locality. Specifically, the areas with strong constraint dissipate stress within the coal sample due to the high strength and stiffness of CFRP, maintaining integrity, whereas the areas with little or no constraint show significant damage, contrasting sharply with the well-preserved regions.

Among them, the CFRP fully confined coal cylinder achieves uniform constraint by setting the net spacing ratio *R* =  0, which effectively limits the lateral deformation of the coal cylinder during axial compression. This enhances shear resistance and stability through the synergistic effect between the CFRP and the coal cylinder. In contrast, the key advantage of CFRP strip-confined coal cylinders lies in the local formation of high-strength support areas, which provide additional support and constraints when the specimen is subjected to shear force, thereby limiting lateral deformation and shear failure. At the same time, the arch effect allows the coal cylinder to transfer part of the pressure to the unconstrained areas during compression, thereby dispersing the load and improving overall bearing capacity.

In summary, CFRP full constraint can significantly enhance the compressive strength and deformation capacity of coal cylinders. However, the use of CFRP strips to constrain coal cylinders also effectively improves their mechanical properties. Compared to full constraint, strip constraint significantly reduces material consumption, thus improving the economic efficiency and cost-effectiveness of the reinforcement process.

### 5.2 Equivalent thickness

**Fig 16:**
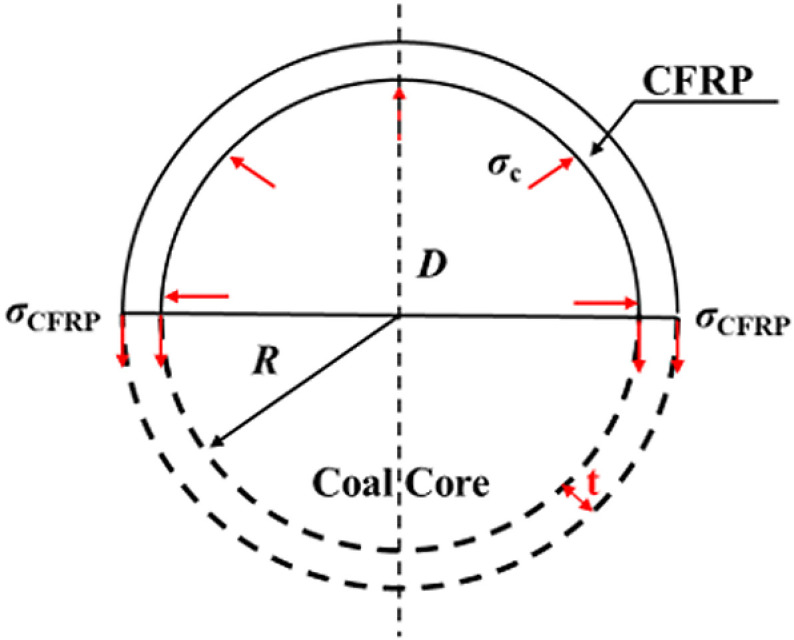
Force balance relationship of CFRP- confined coal cylinders.

Room-and-pillar mining leaves a large number of coal pillars in the goaf, resulting in significant waste of residual coal resources. Based on the work of Zhang et al. [35] and considering the context of this study, it is assumed, according to the principle of equivalent stiffness, that the lateral restraint stiffness *k* provided by CFRP cloth is equivalent to the stiffness *k*_c_ of a hollow coal cylindrical sleeve with a certain thickness *T*_eq_. Considering the underground environment of coal pillars, CFRP constraints can be treated as coal sleeves with a specific thickness. Assuming that the coal cylinder is constrained by a coal sleeve of a certain thickness, the equivalent thickness of the peripheral coal sleeve can be determined by equating the constraint stiffness. The effective lateral restraint provided by CFRP under load significantly increases the overall strength of the coal cylinder. Therefore, CFRP is effectively providing a certain strength constraint stiffness for the coal cylinder [35]. Based on this, a formula is proposed to quantify the constraint stiffness provided by CFRP when it is wound along the hoop direction. The equilibrium relationship of the critical failure force of the CFRP-confined coal cylinder is shown in Fig 16.

Considering the equilibrium conditions in the cross-section of the coal cylinder, the constraint stress acting on the coal cylinder can be calculated as follows:


$$\sigma_{c}=\frac{2\sigma_{CFRP}t}{D}$$ (7)

where, *σ*_c_ is the constraint stress of coal cylinder, MPa; *t* and *D* are the thickness of CFRP and the diameter of coal cylinder, respectively, mm; *σ*_CFRP_ is the circumferential stress of CFRP.

CFRP behaves in a linear elastic manner prior to failure, and its stress-strain relationship can be expressed as follows:


$$\sigma_{CFRP}=\varepsilon_{h,rup}E_{CFRP}$$ (8)

where, *E*_*CFRP*_ is the elastic modulus of CFRP, *ε*_*h,rup*_ is CFRP constrain the circumferential strain of the coal cylinder

Considering the compatibility condition in the inner direction of the coal cylinder section, the circumferential strain of the CFRP is assumed to be equal to the circumferential strain of the coal cylinder.


$$\varepsilon_{c}=\varepsilon_{h,rup}$$ (9)

By substituting Equations (8) and (9) into Equation (7), we can derive the expressions for circumferential stress and circumferential strain:


$$\sigma_{c}=\frac{2tE_{CFRP}}{D}\varepsilon_{c}$$ (10)

The confinement stiffness of the CFRP confinement can be determined by calculating the ratio of circumferential stress to circumferential strain:


$$k=\frac{2tE_{CFRP}}{D}$$ (11)

In order to account for the deterioration effect of the unconstrained region between adjacent strips on the constraint stress, referred to as the ‘arch effect’ assumption [26], it is generally assumed that a parabola with an initial slope of 45° is used as the dividing line between the effective constrained region and the non-effective constrained region. Additionally, the ratio of the area of the effective constrained region to the area of the non-effective constrained region in the middle section of the unconstrained region is used to quantify this deterioration factor, as shown in the formula.


$$k_{v}=\frac{\pi {\left[D-\left(s/2\right)\right]}^{2}/4}{\pi D^{2}/4}={\left(1-\frac{s}{2D}\right)}^{2}$$ (12)

where, *k*_v_ is the effective constraint coefficient.


$$K=k\cdot k_{v}$$ (13)

where, *K* is the constraint stiffness of partially constrained coal cylinder CFRP. According to the Eq. (11), Eq. (12) and Eq. (13), the constraint stiffness corresponding to the CFRP confined coal cylinder can be calculated under different layers and net spacing ratios.

Referring to the stress model of the shaft wall of the cylindrical shaft [36], to simplify the calculation process, the inner diameter of the coal cylinder is set as *a*, and the outer diameter of the hollow coal cylindrical sleeve with equivalent thickness is set as *b*. The elastic solution for the thick-walled hollow coal cylinder under uniform internal radial pressure *q* is derived as:


$$\sigma_{r}=\frac{q}{1-{\left(\frac{a}{b}\right)}^{2}}\left[1-{\left(\frac{a}{r}\right)}^{2}\right]-q$$ (14)

$$\sigma_{\theta }=\frac{q}{1-{\left(\frac{a}{b}\right)}^{2}}\left[1+{\left(\frac{a}{r}\right)}^{2}\right]-q$$ (15)

$$\sigma_{z}=\mu (\sigma_{r}+\sigma_{\theta })$$ (16)

$$\varepsilon_{r}=\frac{1}{E}[\sigma_{r}-\mu (\sigma_{z}+\sigma_{\theta })]$$ (17)

where, *σ*_r_、*σ*_θ_、*σ*_z_、*ε*_r_ are radial stress, tangential stress, vertical stress, radial strain; *r* is the radius of any calculation unit in the cylindrical sleeve of equivalent thick-walled hollow coal, mm; *μ* is the Poisson ‘s ratio of unconstrained coal cylinder; *E* is the elastic modulus of coal cylinder, GPa.

By sorting out Eq. (14) and Eq. (15), we can get：


$$\sigma_{r}=\frac{qa^{2}}{b^{2}-a^{2}}\left[1-{\left(\frac{b}{r}\right)}^{2}\right]$$ (18)

$$\sigma_{\theta }=\frac{qa^{2}}{b^{2}-a^{2}}\left[1+{\left(\frac{b}{r}\right)}^{2}\right]$$ (19)

Substituting the reorganized Eq. (18) and Eq. (19) into Eq. (16), we can get：


$$\sigma_{z}=\frac{2\mu qa^{2}}{b^{2}-a^{2}}$$ (20)

Substituting Eq. (18), Eq. (19) and Eq. (20) into Eq. (17), we can get:


$$\varepsilon_{r}=\frac{1}{E}\cdot \frac{q.a^{2}}{b^{2}-a^{2}}\cdot \left[1-\mu -2\mu^{2}+\left(\mu -1\right){\left(\frac{b}{r}\right)}^{2}\right]$$ (21)

The lateral restraint stiffness provided by the equivalent thickness hollow coal cylindrical sleeve *k*_c_, It can be determined by the ratio of radial stress Eq. (18) to radial strain Eq. (21), and simplified to obtain:


$$k_{c}=\frac{E}{1+\mu }\cdot \frac{b^{2}-r^{2}}{\left[b^{2}-r^{2}(1-2\mu )\right]}$$ (22)

When the calculation unit radius *r* = *a* = 0.5*D*, and *b* = 0.5*D* + *T*_eq_, it can be substituted into Eq. (22)：


$$k_{c}=\frac{E}{1+\mu }\cdot \frac{{\left(0.5D+T_{eq}\right)}^{2}-{\left(0.5D\right)}^{2}}{\left[{\left(0.5D+T_{eq}\right)}^{2}-{\left(0.5D\right)}^{2}(1-2\mu )\right]}$$ (23)

By using the principle of equal stiffness, the Eq. (24) is established：


$$k_{c}=K$$ (24)

It can be derived that *T*_eq_.

In order to further explore the mapping relationship between the equivalent thickness, the clear distance ratio, and the number of CFRP constrained layers, nonlinear fitting analysis is performed. As a result, the equivalent three-dimensional functional relationship that characterizes the different layers of CFRP-constrained coal cylinders under various clear distance ratios is obtained, as shown in Eq. (25):


$$T_{eq}=\frac{\left(0.998-1.945\times R+1.49\times L\right)}{\left(1+0.908\times R-0.221\times L\right)}$$ (25)

**Fig 17:**
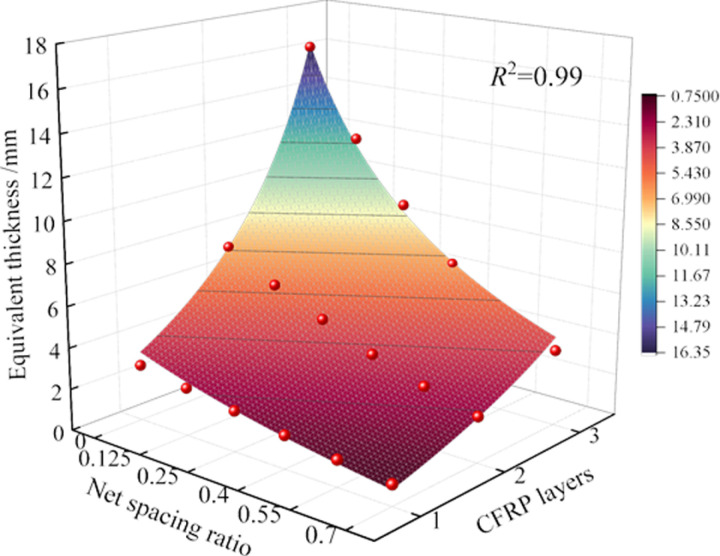
Equivalent thickness three-dimensional surface of CFRP-confined coal cylinders.

The three-dimensional surface diagram of the equivalent thickness of the coal cylinder constrained by different CFRP layers under various clear distance ratios is shown in Fig 17. The fitting accuracy is R² =  0.99, indicating that the fitting effect is remarkable. The results demonstrate that, for the same number of CFRP-constrained layers, the smaller the strip spacing ratio, the greater the equivalent thickness. Therefore, the application of CFRP for the reinforcement of underground coal pillars can effectively improve the recovery rate and has significant potential for broader applications.

**Fig 18:**
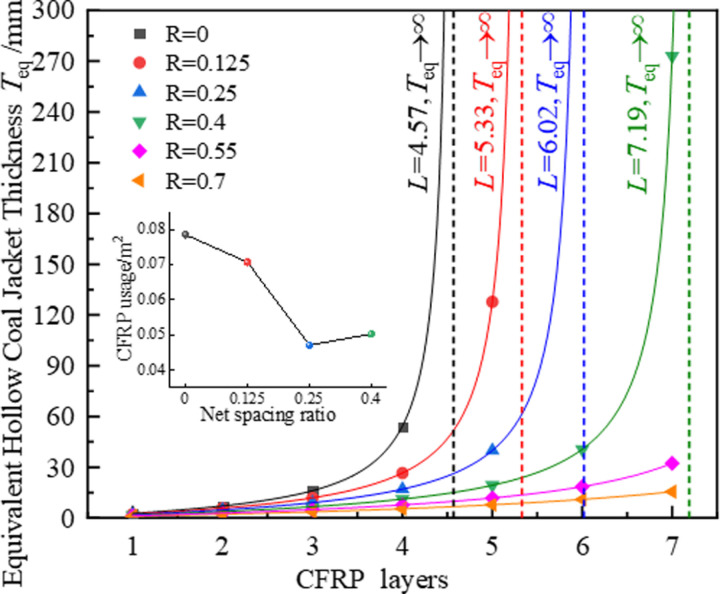
Equivalent thickness evolution curves for CFRP-confined coal cylinders with different net spacing ratios.


Fig 18 shows the curve of the equivalent thickness as a function of the number of CFRP-constrained layers under different net spacing ratios. As the number of CFRP-constrained layers increases, the equivalent thickness curves under each net spacing ratio exhibit a similar development trend. The Rational5 function is used to represent the relationship between the equivalent thickness and the number of CFRP layers:

$$T_{eq}=\frac{p_{1}+p_{2}\cdot L}{1+p_{3}\cdot L+p_{4}\cdot L^{2}}$$ (26)

where, *p*_1_, *p*_2_, *p*_3_, *p*_4_ are function parameters, as shown in Table 4.

**Table 4:** Results of parameters.

*R*	Rational5				R^2^
	*P*_1_	*P*_2_	*P*_3_	*P*_4_	
0	‒0.26523	2.64359	‒0.09224	‒0.02757	0.999
0.125	‒0.07562	2.05858	‒0.13045	‒0.01071	
0.25	‒0.1668	1.92204	‒0.08811	‒0.0129	
0.4	‒0.17517	1.62539	‒0.06961	‒0.00963	
0.55	‒0.1052	1.2731	‒0.06856	‒0.00506	
0.7	‒0.01587	0.94577	‒0.07187	‒0.00147	

Based on the data shown in Fig 18, the net spacing ratio is the core parameter that controls the performance of CFRP-confined coal cylinders, and its change significantly impacts both the amount of CFRP material used and the mechanical response characteristics of the coal cylinders. Specifically, as the net spacing ratio increases from 0 to 0.4, the ideal number of wrapped CFRP layers gradually increases from 4.57 layers (actual number: 5 layers, 0.0785 m²) to 7.19 layers (actual number: 8 layers, 0.0502 m²). During this process, the number of layers increases, while the amount of CFRP material initially decreases and then increases. Notably, when R =  0.25, the number of wrapped layers remains unchanged at 6 layers, while the amount of CFRP material decreases to the lowest value (0.0471 m²).

Building on the in-depth discussion of the influence of the net spacing ratio on the peak strength and ultimate strain of the coal cylinder in Section 3, although increasing the net spacing ratio can reduce the amount of CFRP, there is a threshold beyond which the positive effect on the mechanical properties of the coal cylinder diminishes. When the net spacing ratio is set to 0.25, it not only effectively improves the peak strength and ultimate strain of the coal cylinder, but also achieves a better balance between the amount of CFRP and the improvement in mechanical properties, yielding good economic benefits. Therefore, considering both performance improvement and cost control, the CFRP constraint scheme with a net spacing ratio of 0.25 and a number of wrapped layers of 6 is recommended as the most cost-effective option.

The current research primarily focuses on uniaxial compression tests conducted in the laboratory, and there is a need to consider more complex factors that may arise in real-world engineering applications. Future studies should delve deeper into the mechanisms behind the CFRP strip confinement of coal pillars, exploring how these mechanisms can be optimized for more effective design strategies. Additionally, research could expand to investigate the mechanical behavior of CFRP-reinforced coal pillars under more complex stress states, which better simulate the varied loading conditions found in practical scenarios. Moreover, long-term stability, durability, and the material performance of CFRP reinforcements under cyclic loading and environmental factors such as temperature and humidity should be thoroughly examined to assess their suitability for long-term use in coal mine reinforcement. This broader scope will help to better understand the performance and limitations of CFRP confinement in real-world applications, ultimately leading to more reliable and robust design methods for coal pillar strengthening.

## 6. Conclusion

This study presents an innovative approach to coal pillar reinforcement, highlighting how CFRP reinforcement technology serves as an efficient and cost-effective alternative to traditional methods. By minimizing the use of backfill material, this approach effectively reduces overall costs associated with reinforcement. Its high strength and lightweight characteristics streamline the construction process, enhance engineering efficiency, and lower environmental impact, positioning it as a more sustainable reinforcement solution. To examine the influence of the net spacing ratio of CFRP strips and the number of CFRP layers on coal columns, a total of 39 CFRP-constrained coal column specimens were subjected to experimental investigation. Based on the experimental study and theoretical analysis of the axial compression performance of CFRP-constrained coal columns, the following conclusions can be drawn:

1. Laboratory experiments demonstrate that the net spacing ratio and the number of CFRP layers are crucial factors influencing the axial compression performance of coal cylinders. As the net spacing ratio decreases and the number of CFRP layers increases, the peak strength and deformation capacity of the coal cylinder are significantly enhanced. By optimizing the net spacing ratio of CFRP strips, both the bearing capacity and stability of coal cylinders can be significantly improved.
2. The net spacing ratio and the number of CFRP layers significantly affect the failure mode of the coal cylinder. CFRP strips play a critical role in the process of constraining coal cylinders, and their fracture and failure are the primary controlling factors for the failure of coal cylinders. From the perspective of failure mode, the failure of CFRP-partially confined coal cylinders primarily occurs due to the fracture of the CFRP strip in the middle and the crushing of local areas of the coal cylinder. A smaller net spacing ratio and a greater number of CFRP layers can more effectively restrict the lateral expansion of the coal cylinder and reduce the extent of damage.
3. The failure of the coal cylinder is an energy dissipation process, and its energy dissipation ratio follows an ‘N’-shaped trend with increasing strain, reflecting the different stages of the coal cylinder’s behavior, from crack closure and elastic energy accumulation to final failure. Unconstrained coal cylinders are more prone to impact damage due to their inherent brittle characteristics, and the confinement provided by CFRP can delay this process, enhancing the energy absorption capacity of the coal cylinders. This significantly contributes to the safe production of coal mine engineering.
4. Considering the characteristics of the equivalent thickness evolution curve, peak strength, and ultimate strain of CFRP-confined coal cylinders under different spacing ratios, and taking into account economic cost factors such as CFRP usage, the CFRP constraint method with a net spacing ratio of 0.25 and a number of wrapped layers of 6 is ultimately determined to be the most cost-effective option.

## Supporting information

S1 Data Raw data of figures. (ZIP)
